# Utility of Cardiovascular Magnetic Resonance-Derived Wave Intensity Analysis As a Marker of Ventricular Function in Children with Heart Failure and Normal Ejection Fraction

**DOI:** 10.3389/fped.2017.00065

**Published:** 2017-04-03

**Authors:** Hopewell N. Ntsinjana, Robin Chung, Paolo Ciliberti, Vivek Muthurangu, Silvia Schievano, Jan Marek, Kim H. Parker, Andrew M. Taylor, Giovanni Biglino

**Affiliations:** ^1^Centre for Cardiovascular Imaging, Institute of Cardiovascular Science, University College London, London, UK; ^2^Cardiorespiratory Division, Great Ormond Street Hospital for Children, NHS Foundation Trust, London, UK; ^3^Department of Paediatrics, Paediatric Cardiology Division, CH Baragwanath Academic Hospital, University of the Witwatersrand, Johannesburg, South Africa; ^4^Department of Pediatric Cardiology and Cardiac Surgery, Pediatric Hospital “Bambino Gesù”, Rome, Italy; ^5^Department of Bioengineering, Imperial College, London, UK; ^6^School of Clinical Sciences, Bristol Heart Institute, University of Bristol, Bristol, UK

**Keywords:** systolic function, diastolic function, wave intensity analysis, ventricular mechanics, cardiovascular magnetic resonance

## Abstract

**Objective:**

This study sought to explore the diagnostic insight of cardiovascular magnetic resonance (CMR)-derived wave intensity analysis to better study systolic dysfunction in young patients with chronic diastolic dysfunction and preserved ejection fraction (EF), comparing it against other echocardiographic and CMR parameters.

**Background:**

Evaluating systolic and diastolic dysfunctions in children is challenging, and a gold standard method is currently lacking.

**Methods:**

Patients with presumed diastolic dysfunction [*n* = 18; nine aortic stenosis (AS), five hypertrophic, and four restrictive cardiomyopathies] were compared with age-matched control subjects (*n* = 18). All patients had no mitral or aortic incompetence, significant AS, or reduced systolic EF. *E*/*A* ratio, *E*/*E*′ ratio, deceleration time, and isovolumetric contraction time were assessed on echocardiography, and indexed left atrial volume (LAVi), acceleration time (AT), ejection time (ET), and wave intensity analyses were calculated from CMR. The latter was performed on CMR phase-contrast flow sequences, defining a ratio of the peaks of the early systolic forward compression wave (FCW) and the end-systolic forward expansion wave (FEW).

**Results:**

Significant differences between patients and controls were seen in the *E*/*E*′ ratio (8.7 ± 4.0 vs. 5.1 ± 1.3, *p* = 0.001) and FCW/FEW ratio (2.5 ± 1.6 vs. 7.2 ± 4.2 × 10^−5^ m/s, *p* < 0.001), as well as—as expected—LAVi (80.7 ± 22.5 vs. 51.0 ± 10.9 mL/m^2^, *p* < 0.001). In particular, patients exhibited a lower FCW (2.5 ± 1.6 vs. 7.2 ± 4.2 × 10^−5^ m/s, *p* < 0.001) in the face of preserved EF (67 ± 11 vs. 69 ± 5%, *p* = 0.392), as well as longer isovolumetric contraction time (49 ± 7 vs. 34 ± 7 ms, *p* < 0.001) and ET/AT (0.35 ± 0.04 vs. 0.27 ± 0.04, *p* < 0.001).

**Conclusion:**

This study shows that the wave intensity-derived ratio summarizing systolic and diastolic function could provide insight into ventricular function in children, on top of CMR and echocardiography, and it was here able to identify an element of ventricular dysfunction with preserved EF in a small group of young patients.

## Introduction

Assessment of systolic and diastolic dysfunctions in pediatric patients poses difficulties due to the lack of a “gold standard” diagnostic biomarker. Currently, ventricular function, in particular diastolic dysfunction, is best assessed using catheter-based techniques, though these are invasive in nature and present technical limitations (1). Echocardiography offers a non-invasive alternative, including detection of early myocardial dysfunction before any detectable change in ejection fraction (EF) (2), but it also presents limitations, partly due to the fact that existing normal echocardiographic values have too broad a range in the pediatric population (3).

In the adult population, up to 50% of elderly patients with heart failure have a normal ejection fraction (HFNEF) (4). This entity has also been observed in the pediatric literature, with several pediatric cardiac diseases that can present with diastolic dysfunction in the face of a normal EF. These include congenital aortic stenosis (AS) (5), hypertrophic cardiomyopathy (HCM) (6), and restrictive cardiomyopathy (RCM) (6), where young patients had findings consistent with diastolic dysfunction and normal EF. With the current diagnostic tests having potential limitations in children (and also in adults), we sought to assess a novel index obtained from wave intensity analysis [derived non-invasively from cardiovascular magnetic resonance (CMR) data] in children with a high likelihood of diastolic dysfunction but normal EF, in order to gather better insight into their ventricular function.

Wave intensity represents energy flux per unit area carried by waves traveling in the cardiovascular network (7). This hemodynamic quantity carries information on interactions between the heart and the vasculature, i.e., ventriculoarterial coupling (8–10). Early work in this context suggested the potential of this method to provide insight into ventricular filling mechanics and speculated that it could help in the detection and characterization of diastolic dysfunction (11). This article aims to explore the diagnostic insight of CMR-derived wave intensity analysis in a group of children with preserved EF and chronic diastolic dysfunction, as a way to better study systolic dysfunction in patients with diastolic dysfunction.

## Materials and Methods

### Patient Population

We retrospectively identified a total of 31 patients who had undergone both CMR and detailed echocardiogram and were labeled to have presumed diastolic dysfunction. The datasets from these patients were then subjected to strict inclusion criteria for further analysis with wave intensity, namely:

(a) Dilated left atrium (LA) on CMR and echocardiogram (*Z*-score ≥ +2.0)
(b) Normal EF ≥ 55% on CMR
(c) No mitral valve stenosis or regurgitation
(d) No significant AS (*V*_max_ gradient < 30 mmHg) or aortic regurgitation.

Eighteen subjects fitted the criteria, whereby no study patient had mitral or aortic incompetence, significant AS or reduced systolic EF:

- Nine patients had congenital AS—four patients had neonatal balloon aortic valvuloplasty followed by Ross procedure (pulmonary autograft aortic valve replacement and pulmonary homograft procedure) to treat residual AS and five patients had neonatal surgical valvotomy, followed by Ross procedure.
- Five patients had HCM with the evidence of dilated LA on CMR or echocardiography, normal EF on CMR (>55%), no left ventricular outflow tract obstruction, and no history of myomectomy or alcohol ablation.
- Four patients had idiopathic RCM based on high end-diastolic pressures (EDP) on previous catheterization, echocardiographic features reported as restrictive physiology (dilated LA and Doppler evidence of restriction), and normal EF (>55%).

Eighteen healthy age-matched controls with no current or past evidence of phenotypic cardiovascular disorders served as our control subjects.

### Echocardiographic Data

**Figure 1:**
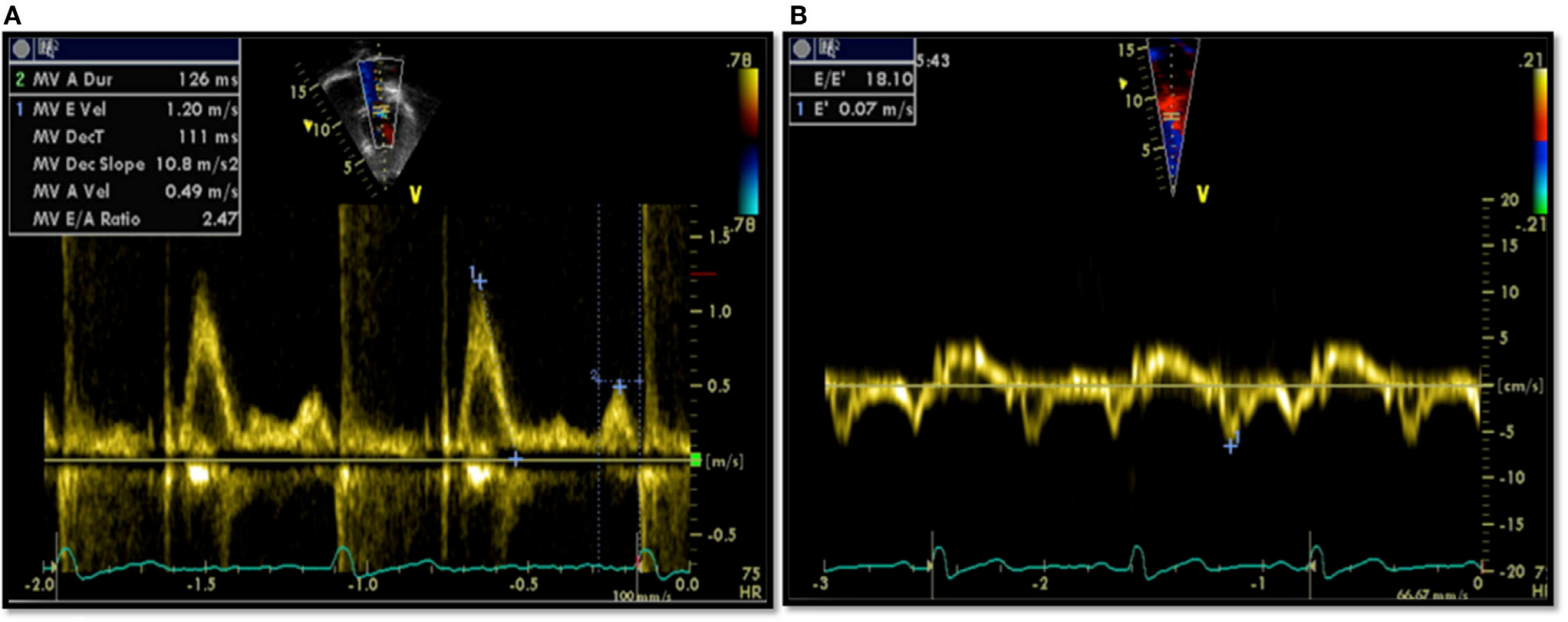
**Echocardiographic data from a patient showing diastolic dysfunction: (A) trans-mitral Doppler *E*/*A* = 2.4, deceleration time = 111 ms; (B) tissue Doppler imaging *E*′ = 0.07, a high *E*/*E*′ ratio of 18.1 signifying diastolic dysfunction**.

All patients had an echocardiographic examination as a part of their assessment. Diastolic function assessment included pulsed Doppler of the mitral inflow and tissue Doppler imaging (TDI). All measurements of diastolic variables were retrospectively re-measured by a single echocardiographer. Conventional pulsed Doppler indices of diastolic function, peak early (*E*) and late (*A*) diastolic trans-mitral velocities, *E*/*A* ratio, and E-wave deceleration time (DT) were measured from the spectral Doppler signal of the mitral valve inflow, together with the isovolumetric contraction time (IVCT). Pulsed-wave TDI velocities were obtained from the septal mitral annulus (apical four-chamber view). TDI measurements for each myocardial segment included peak early diastolic velocity (*E*′) and peak late diastolic velocity (*A*′). Only tracings that demonstrated a clear *E*′ were used. Each TDI velocity was measured on three consecutive cardiac cycles and averaged for the analysis. The ratio of peak early diastolic mitral inflow velocity and early septal TDI velocity (*E*/*E*′) was calculated. Examples of echocardiographic data are provided in Figure 1.

### CMR Data

Cardiovascular magnetic resonance data were acquired with a 1.5-T scanner (Avanto; Siemens Medical Solutions, Erlangen, Germany). Flow quantification was performed using phase-contrast sequences, with through-plane flow data acquired with the use of retrospective cardiac gating. Appropriate velocity-encoding values were set ~250 cm/s for through-plane flow quantification. Indicative parameters for the flow phase-contrast sequences were TR/TE = 27/3 ms, flip angle = 30°, and pixel size = 1.25 × 1.25, with a field of view = 240 × 320 or smaller. Acquisition parameters were adapted if necessary to optimize the quality of the scan for each case. Retrospectively gated, balanced, steady-state free precession cine images were acquired in the vertical long-axis (two-chamber), four-chamber, and the short-axis covering the entirety of both ventricles (9–12 slices). Myocardial late gadolinium enhancement for tissue characterization was performed in the long- and short-axis planes, using inversion recovery prepared gradient recalled echo sequence 10–15 min after injection of gadolinium (Dotarem^®^, gadoterate meglumine, Gd-DOTA, 0.1 mmol/kg; Guerbet, Paris, France). Inversion time was adjusted (250–350 ms) in order to null the normal viable myocardium. All postprocessing was carried out using in-house written plug-ins (OsiriX; Pixmeo, Geneva, Switzerland).

**Figure 2:**
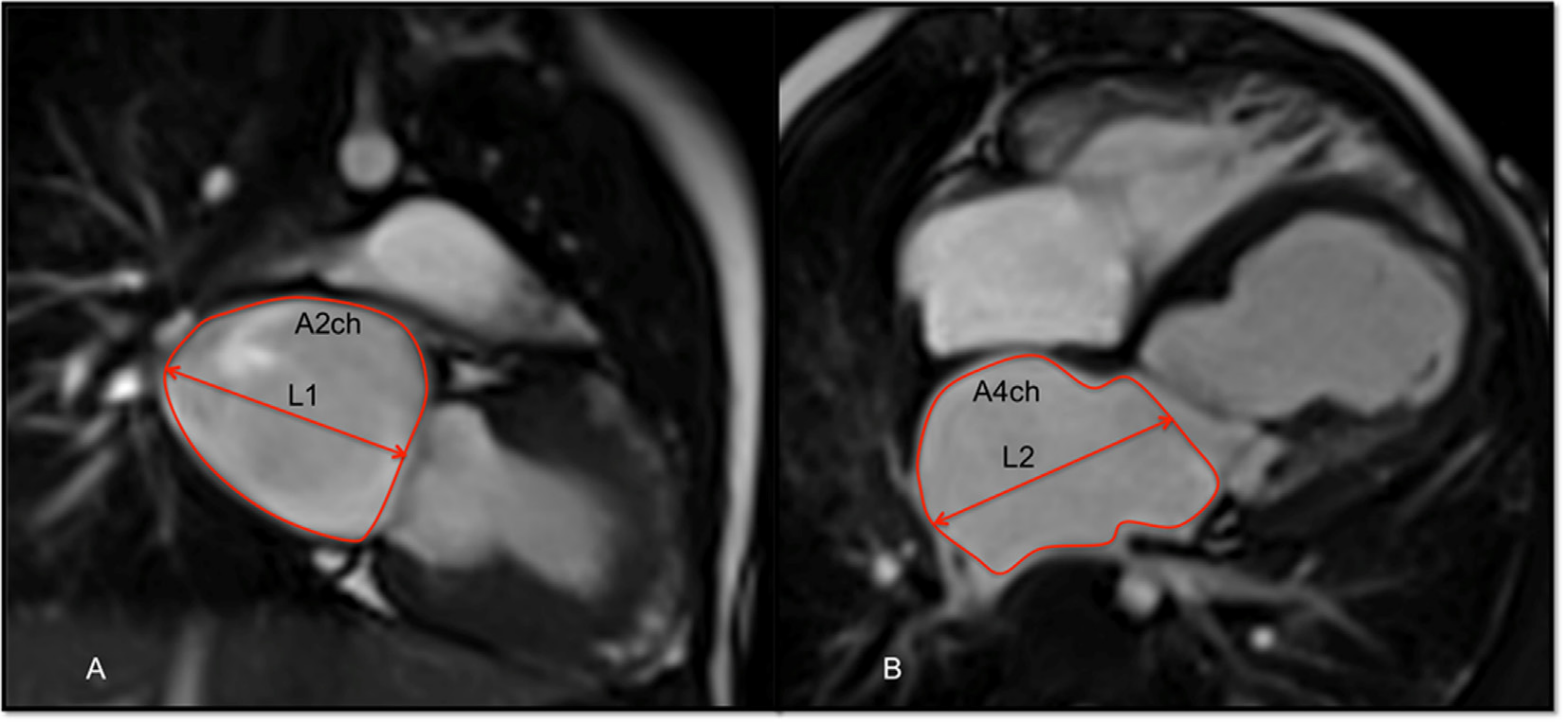
**Balanced-SSFP cine images of: (A) two-chamber (2ch) view and (B) four-chamber (4ch) at end systole showing measurement of the left atrial volume (LAV) by the biplane area–length method**. The atrial endocardial border was traced to delineate left atrium (LA) area excluding the pulmonary veins, LA appendage, and mitral valve recess. LA volume was measured as reported in the Section “Materials and Methods.”

Left ventricular end-diastolic and end-systolic volumes were measured and indexed for BSA (i). The stroke volume, EF, and cardiac output were derived from these measurements. On top of the functional data, LA area and length were also measured from the two-chamber and four-chamber views, respectively, at the end of ventricular systole. The LA area was obtained by tracing its endocardial border excluding the pulmonary veins, LA appendage, and mitral valve recess. These parameters were used to calculate LA volume (LAV) using the formula (12, 13):

$$\text{LAV}=\frac{8(\text{A}2\text{ch})(\text{A4ch})}{3\pi \text{L}}$$

where A2ch is the area of the two-chamber view, A4ch is the area of the four-chamber view, and *L* is the shorter of the two LA length measurements (L2Ch and L4Ch) from these views (Figure 2).

The aortic velocity waveforms were used to compute peak velocity, acceleration time (AT), and ejection time (ET).

### Wave Intensity Analysis

Wave intensity carries important information on ventriculoarterial interactions as well as time-domain information on wave reflections (7). By formulating wave intensity in terms of area (*A*) and velocity (*U*) differentials, i.e. *dI* = *dUd*ln*A*, as opposed to the traditional pressure-based formulation, i.e., *dI* = *dPdU*, the analysis can be performed non-invasively based on CMR phase-contrast acquisitions. Wave intensity information was derived from CMR data using a previously proposed methodology (14). The image processing was carried out using an in-house written plug-in (OsiriX), whereby images are semiautomatically segmented to extract the *A* and *U* signals from the ascending aortic flow sequence and these are then combined to compute *dI*.

**Figure 3:**
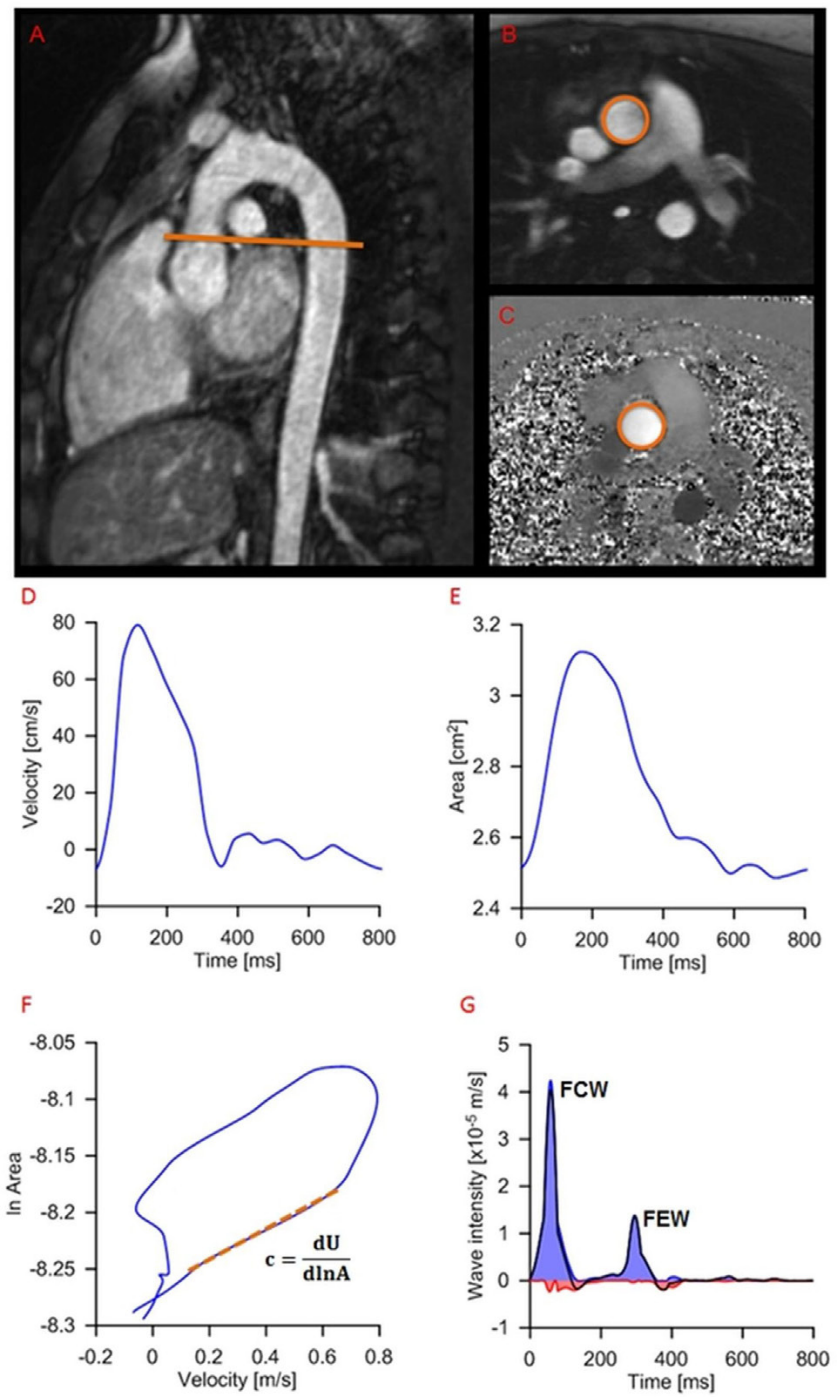
**Example of wave intensity analysis methodology from a patient, showing positioning of the slice for flow assessment (A) and modulus (B) and phase (C) images of the phase-contrast acquisition**. Aortic velocity **(D)** and area **(E)** are semiautomatically extracted and combined for wave speed calculation **(F)** and wave intensity analysis **(G)**: *c*, wave speed; *A*, area; *U*, aortic velocity; FCW, forward compression wave (early systole); FEW, forward expansion wave (end systole).

The following two dominant waves are identified in a typical *dI* pattern: a forward compression wave (FCW) at systolic ejection and a forward expansion wave (FEW) at end systole (Figure 3). Previous work has linked FCW with ventricular *dP*/*dt* and FEW with diastolic time relaxation constant (*τ*), suggesting *dI* as a clinically useful parameter for concurrently assessing LV systolic and early diastolic performance (15). Not only systolic and diastolic dysfunctions are unlikely to occur in isolation, but furthermore a desirable test for patients with heart failure has been suggested to be a non-invasive assessment of systolic and diastolic left ventricular functions together, not to uncouple systolic from diastolic function (16). For this reason, a single, non-dimensional parameter is proposed here and calculated as the ratio of *dI* peaks, i.e., =FCW/FEW, as an indicator of ventricular function. The rationale for choosing this parameter was based on the observation that systolic and diastolic dysfunctions often coexist, and the combined ratio would likely be more informative of cardiac dysfunction than systolic/diastolic measures alone (17, 18).

### Data Analysis

All data processing was carried out in Matlab (MathWorks, Natick, MA, USA). Healthy controls and patients were compared for the following parameters: *E*/*A, E*/*E*′, DT, and IVCT from echocardiography; EF, indexed left atrial volume (LAVi) and AT/ET from CMR; and FCW/FEW. Aortic distensibility (*D*) was inferred from wave speed (*c*) according to the Bramwell–Hill formula, *c*^2^ = 1/ρ*D*, with ρ = density of blood and *c* estimated as a part of the CMR-based wave intensity analysis.

### Statistical Analysis

Statistical analysis was carried out with commercial software (SPSS v.22; SPSS Inc., Chicago, IL, USA). Data are presented as mean ± SD. Comparisons of continuous variables of unpaired samples between groups (controls vs. patients) were performed using unpaired two-tailed Student’s *t*-test, or Mann–Whitney *U* test for non-parametric data. Linear correlation among different variables was assessed using the Pearson *r* coefficient. A value of *p* < 0.05 was regarded as statistically significant.

## Results

Baseline characteristics, CMR, echocardiography, and wave intensity data between patients and healthy control subjects are presented in Table 1. Baseline characteristics showed that patients were age-matched with healthy controls (13.6 ± 4.5 vs. 14.9 ± 2.2 years, *p* = 0.259). There were no blood pressure differences between the two groups.

**Table 1:** **Summary of demographic, echocardiography, CMR, and wave intensity data for patients and healthy controls groups**.

Variables	Patients (*n* = 18)	Controls (*n* = 18)	*p*-Value
**Demographic data**			
Age (years)	13.6 ± 4.5	14.9 ± 2.2	0.259
BSA (m^2^)	1.4 ± 0.4	1.7 ± 0.4	0.005*
Sex (F/M)	7/11	4/14	0.04*
HR (bpm)	78 ± 12	72 ± 12	0.157
DBP (mmHg)	62 ± 11	62 ± 10	0.940
SBP (mmHg)	104 ± 14	106 ± 10	0.520
**Echocardiography data**			
*E*/*A*	2.3 ± 1.1	2.5 ± 1.2	0.625
Deceleration time (ms)	137.0 ± 53.1	154.3 ± 48.5	0.307
*E*/*E*′	8.7 ± 4.0	5.1 ± 1.3	0.001*
IVCT (ms)	49 ± 7	34 ± 7	<0.001*
**CMR data**			
LV ESVi (mL/m^2^)	23.0 ± 9.5	23.6 ± 6.7	0.817
LV EDVi (mL/m^2^)	68.4 ± 11.9	75.2 ± 12.6	0.099
iSV (mL/m^2^)	43 ± 10	52 ± 8	0.010*
LV ejection fraction (%)	67 ± 11	69 ± 5.0	0.392
Indexed left atrial volume (mL/m^2^)	80.7 ± 22.5	51.0 ± 10.9	<0.001*
Peak U (cm/s)	62 ± 16	90 ± 14	<0.001*
Acceleration time (AT) (ms)	117 ± 21	92 ± 15	<0.001*
Ejection time (ET) (ms)	332 ± 36	341 ± 21	0.334
AT/ET	0.35 ± 0.04	0.27 ± 0.04	<0.001*
**WIA data**			
Distensibility (×10^−3^ 1/mmHg)	4.3 ± 3.7	6.5 ± 4.3	0.116
Wave speed (m/s)	8.2 ± 7.2	5.3 ± 2.7	0.115
Peak forward compression wave (FCW) (x10^-5^ m/s)	2.5 ± 1.6	7.2 ± 4.2	<0.001*
Peak forward expansion wave (FEW) (×10^−6^ m/s)	7.8 ± 4.2	6.9 ± 3.9	0.551
FCW/FEW	3.7 ± 2.9	12.7 ± 7.9	<0.001*

*p < 0.05.

### Echocardiographic Evaluation

Indices of conventional pulsed-wave Doppler across the mitral valve and TDI are listed in Table 1. There was no significant difference of *E*/*A* ratio and DT between patients and healthy controls. *E*/*E*′ showed a statistically significant difference between patients and controls (8.7 ± 4.0 vs. 5.1 ± 1.3 *p* = 0.001). IVCT was significantly longer in patients compared to controls (49 ± 7 vs. 34 ± 7 ms, *p* < 0.001).

### CMR Evaluation

Left ventricular functional assessment showed no significant differences of the indexed volumes (LV ESVi and LV EDVi) and EF (67 ± 11 vs. 69 ± 5%, *p* = 0.392) between patients and controls. The LAVi, as expected, was significantly larger in patients with presumed diastolic dysfunction compared to healthy controls (80.7 ± 22.5 vs. 51.0 ± 10.9 mL/m^2^, *p* < 0.001, Table 1). On tissue characterization imaging, four AS patients and one patient with idiopathic RCM displayed endomyocardial fibroelastosis (EFE). Furthermore, patients exhibited lower peak aortic velocity, as well as significantly longer AT and similar ET, resulting in AT/ET = 0.35 ± 0.04 in patients and AT/ET = 0.27 ± 0.04 in controls (*p* < 0.001).

### Wave Intensity Analysis

Peak FCW was significantly lower in patients compared with normal healthy controls (2.5 ± 1.6 vs. 7.2 ± 4.2 × 10^−5^ m/s, *p* < 0.001), while peak FEW was not significantly different (7.8 ± 4.2 vs. 6.9 ± 3.9 × 10^−6^ m/s, *p* = 0.551). The ratio FCW/FEW was thus significantly lower in patients (3.7 ± 2.7 vs. 12.7 ± 7.9, *p* < 0.001). Pearson correlation of the FCW/FEW with well-known echocardiographic and CMR biomarkers of diastolic dysfunction is shown in Table 2. The FCW/FEW ratio had a significant moderate negative correlation with *E*/*E*′ (*r* = −0.325, *p* = 0.027) and a significant negative correlation with presence of EFE (*r* = −0.343, *p* = 0.020), while the correlation with LAVi was just above the significance threshold (*r* = −0.264, *p* = 0.060). Furthermore, a significant negative correlation was found between FCW and IVCT measured from echocardiography (*r* = −0.459, *p* = 0.012) and between FCW and AT/ET derived from CMR aortic velocity data (*r* = −0.386, *p* = 0.018).

**Table 2:** **Values of Pearson’s coefficient (*r*) and *p* values for correlations between forward compression wave/forward expansion wave ratio and other parameters**.

Variables	Pearson’s (*r*)	*p*-Value
Age	0.005	0.487
SBP	0.047	0.393
DBP	0.156	0.181
*E*/*A*	0.163	0.171
*E*/*E*′	−0.325	0.027*
Deceleration time	0.028	0.473
LV ejection fraction	0.226	0.093
Endomyocardial fibroelastosis	−0.343	0.02*
Indexed left atrial volume	−0.264	0.060
Distensibility	0.618	<0.001*

*p < 0.05.

## Discussion

In this study, we demonstrate the diagnostic potential of a novel, wave intensity analysis biomarker, derived from CMR flow data. Importantly, the wave intensity data (i.e., reduced FCW) provided insight into an element of load-independent systolic dysfunction in children with normal EF and chronic diastolic dysfunction. This observation was corroborated by prolonged IVCT on the face of normal diastolic blood pressure; however, IVCT on its own is limited by the fact that invasive pressure data is needed in order to comment on systolic function. The systolic impairment was further confirmed by observing aortic velocity waveforms, whereby peak velocity is significantly lower in patients who concurrently exhibit a longer AT. This indicates that peak aortic acceleration was lower, i.e., lower inotropy (19). Overall, FCW could then be a non-invasive marker for load-independent systolic dysfunction in the face of normal EF.

We have shown that the FCW/FEW ratio provided insight in the physiology of patients with chronic diastolic dysfunction despite normal EF when compared with more conventional non-invasive parameters. Such a non-invasive measurement may prove particularly useful in the pediatric population, as diagnostic tools that are adequate for use in adult populations for the diagnosis of diastolic dysfunction fail to do so in pediatric patients, including echocardiographic parameters routinely used in the clinic (3).

Potential confounders for abnormal diagnostic tests were purposefully eliminated in this study. All patients with mitral valve disease, aortic incompetence, or residual stenosis and those with reduced LV EF were excluded in an attempt to make the study group more homogenous. Our primary objective was to test the potential of the wave intensity data for assessment of sub-clinical ventricular dysfunction. There was no difference in EF between patients and control subjects but significantly increased LAVi in the patient group. This is important as there is a significant relationship between increased LAVi and elevated LV EDP as well as echocardiographic indices of diastolic dysfunction, as shown in adult patients (20, 21). We presume therefore that the abnormally increased LAVi in the diseased subjects in our study is due to chronic diastolic dysfunction, an entity previously shown in pediatric echocardiographic studies (13). The combination of these abnormal and normal measured parameters then gave us this study cohort with normal EF in the presence of diastolic dysfunction.

Our results agree with the literature with regard to echocardiographic parameters in children in that only the *E*/*E*′ was significantly different between patients and healthy control subjects (3, 22, 23). As a proof of principle, the new biomarker was then correlated with the established diagnostic tests for diastolic dysfunction, correlating with the two reliable indicators of diastolic dysfunction, namely *E*/*E*′ and LAVi. An even stronger correlation was found between FCW/FEW and EFE. EFE was detected in 28% of the patients with presumed diastolic dysfunction, 80% of whom had congenital AS as a primary cardiac diagnosis. Physiologically, EFE renders the LV less distensible, thus potentially causing further impairment of diastolic filling (24, 25). Though the majority of our patient group did not have overt heart failure, many of HFNEF has been attributed to LV remodeling associated with concentric hypertrophy and increased end-diastolic volume, hence increased LV stiffness (26, 27). LV systolic and diastolic dysfunctions have long been known to induce impaired relaxation; this has been proven to be true by a myocardial performance index previously proposed by Tei et al. (18), showing a positive correlation between myocardial performance index and *τ*. The Tei index is, however, limited by pseudonormalization in the face of HFNEF and largely susceptible to activation delay (28, 29). Instead, the wave intensity biomarker could be used to complement and/or augment already existing parameters in those patients with HFNEF. From a methodological perspective, this postprocessing methodology does not impinge on scan time (i.e., does not require additional sequences acquisition) and has been semi-automated to facilitate the analysis.

### Limitations and Future Directions

The first limitation is the small number of patients studied, partly due to strict inclusion criteria adopted to avoid clinical confounders. Second, this study was performed on patients with presumed rather than definitive diastolic dysfunction due to no pressure–volume loop data being available as a reference standard; however, we used acceptable non-invasive surrogates of diastolic dysfunction. Third, out-of-plane motion in CMR flow sequences may represent a problem for aortic area measurements. However, this movement has been quantified in the range of <1 cm (30, 31), and assuming no significant tapering in the region of the ascending aorta and constant regional wall properties, area measurements should not be substantially affected. Finally, the diagnostic potential of the proposed wave intensity ratio should be tested in a larger cohort of prospective patients and we intend to carry this out in the near future.

## Conclusion

This study suggests a novel, non-invasive biomarker for assessing sub-clinical ventricular dysfunction in pediatric patients, based on CMR-derived wave intensity analysis, with diagnostic capabilities that appeared to perform better that the standard available parameters. This parameter could be easily implemented in routine CMR examinations (in both children and adults), providing additional and complementary information on combined systolic and diastolic performances.

## Ethics Statement

Informed consent for the use of imaging data was obtained from all parents of patients who were imaged as a part of the patient clinical follow-up. The study was carried out in accordance with the ethical guidelines of the 1975 Declaration of Helsinki, as reflected in prior approval by the institutional and research ethics committee.

## Author Contributions

HN, RC, PC, VM, SS, JM, KP, AT, and GB designed the study, contributed to the data acquisition, analysis, and data interpretation; drafted the article; contributed to the data acquisition, analysis, and interpretation of results and revised critically the article. All authors read and approved the final article.

## Conflict of Interest Statement

The authors declare that the research was conducted in the absence of any commercial or financial relationships that could be construed as a potential conflict of interest.
